# GNAS1 and PHD2 Short-interfering RNA Support Bone Regeneration in Vitro and in an in Vivo Sheep Model

**DOI:** 10.1007/s11999-012-2475-4

**Published:** 2012-07-26

**Authors:** Carmen N. Ríos, Roman J. Skoracki, Anshu B. Mathur

**Affiliations:** 1 Department of Bioengineering, University of Pittsburgh, Pittsburgh, PA USA; 2 Tissue Regeneration and Molecular Cell Engineering Laboratories, Department of Plastic Surgery, The University of Texas M. D. Anderson Cancer Center, 1515 Holcombe Boulevard, Unit 602, Houston, TX 77030-1402 USA

## Abstract

**Background:**

Our ability to guide cells in biomaterials for in vivo bone repair is limited and requires novel strategies. Short-interfering RNA (siRNA) allows the regulation of multiple cellular pathways. Core binding factor alpha 1 (Cbfa1) and hypoxia-inducible factor 1 (HIF-1) pathways can be modulated to direct bone formation via siRNA against guanine nucleotide-binding protein alpha-stimulating activity polypeptide 1 (siGNAS1) and prolyl hydroxylase domain-containing protein 2 (siPHD2), respectively.

**Questions/Purposes:**

We determined whether the administration of siGNAS1 and siPHD2 in mesenchymal stem cells (MSCs) promotes osteogenic phenotype, the dose-dependent effects of siGNAS1 on MSC differentiation to osteogenic phenotype, and whether the two siRNAs promote bone formation in vivo.

**Methods:**

siRNAs were administered to MSCs at Day 0, and protein expression of bone-specific markers was assessed at Days 1, 2, and 4 (n = 3/group/time point). In an in vivo model using seven sheep, chambers containing silk fibroin-chitosan (SFCS) scaffolds with siRNA were implanted over the periosteum and harvested at Days 7, 21, 36, and 70 (n = 4/group/time point, except at Day 70 [n = 2]) to assess bone formation.

**Results:**

siGNAS1 promoted collagen I and osteopontin expression, whereas siPHD2 had no effect in vitro. Dose-dependent effects of siGNAS1 on ALP expression were maximal at Day 1 for 10 μg/mL and Day 4 for 100 μg/mL. In vivo, by Day 70, mean bone volume increased compared to Day 7 for siGNAS1-SFCS (47.8 versus 1.8 mg/mL) and siPHD2-SFCS (61.3 versus 1.5 mg/mL).

**Conclusions:**

Both siPHD2 and siGNAS1 support bone regeneration in vivo, whereas only siGNAS1 regulates bone phenotype in MSCs in vitro.

**Clinical Relevance:**

While the use of autologous tissue is limited for reconstructing critical-sized defects, the development of biomaterial-based approaches to promote bone formation may abrogate some of those limitations.

## Introduction

Regenerative medicine has the potential to revolutionize reconstructive approaches for critical-sized bone defects by providing prefabricated tissue with patient-specific geometry while minimizing donor tissue requirements [4, 13, 17]. Repair of critical-sized bone defects with cadaver-derived bone graft, biomaterials such as titanium, hydroxyapatite, and ceramics, autologous vascularized tissue transfers, or prosthetic rehabilitation have limitations and thus the use of biodegradable bone-conducting biomaterials is being explored [13]. Ectopically grafted periosteum and silk fibroin-chitosan (SFCS) biomaterial reportedly regenerate bone in an in vivo sheep model [4, 7, 8, 16]. These studies suggest an approach to bone repair by prefabricating biomaterial-based transferable bone tissue flaps is a potential alternative.

Stimulation of cellular processes for tissue-engineering applications has focused primarily on delivery of proteins or nucleic acids, antisense oligonucleotides, adenoviral-based delivery, and hammerhead ribozymes, which have been used to modulate the expression of proteins [6, 10]. These methods, however, have been plagued with oligonucleotide stability [10] or nonspecific global suppression [6]. Short-interfering RNA (siRNA) is a gene-silencing process involving the introduction of 21- to 25-nucleotide double-stranded RNA into a cell that results in the degradation of the complementary mRNA via a multiple enzyme complex called RNA-induced silencing complex [3]. A short nucleotide can be introduced to target multiple pathways/factors, thus providing a means to guide diverse and interlinked cellular processes simultaneously [3]. siRNA that targets the hypoxia pathway turns on the angiogenic protein expression via the hypoxia-inducible factor 1 (HIF-1) and siRNA that targets the bone differentiation pathway turns on the core binding factor alpha 1 (Cbfa1) pathway to activate expression of bone differentiation proteins.

**Fig. 1A–B:**
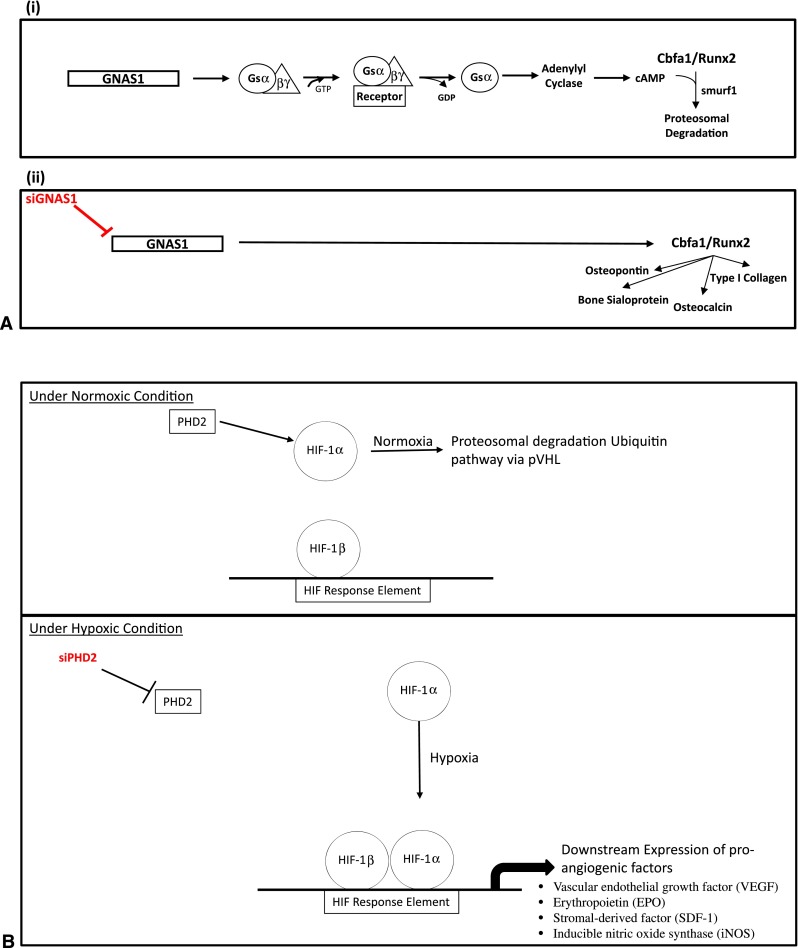
(**A**) A schematic of the Cbfa1 pathway shows the action of siGNAS1. The blocking of the GNAS1 gene via siGNAS1 results in the activation of the Cbfa1 pathway and expression of proteins such as osteopontin, collagen I, and others. GTP = guanosine triphosphate; GDR = guanosine diphosphate; cAMP = cyclic adenosine monophosphate. (**B**) A schematic shows the hypoxia pathway regulated by HIF-1 (HIF-1α and HIF-1β) and targeted to regulate angiogenesis by blocking of PHD2 via siPHD2, which allows the binding of HIF-1α to HIF-1β and expression of VEGF, EPO, SDF-1, and iNOS.

Cbfa1 is a transcript factor important in differentiation of bone precursor cells into osteoblasts and subsequent bone formation. The activity of Cbfa1 is regulated by the α chain of the heterotrimeric G protein, Gsα, and is transcribed by the guanine nucleotide-binding protein (G protein) alpha-stimulating activity polypeptide 1 (GNAS1) gene (Fig. 1A) [6, 10]. Expression of Gsα inhibits osteogenic differentiation by means of proteolytic degradation of Cbfa1. The downregulation of the GNAS1 gene via siRNA leads to the increased expression of Cbfa1 that would stimulate the production of bone-differentiating proteins such as osteopontin, collagen I, and osteocalcin [3, 15].

HIF-1 is a transcription factor and a heterodimer composed of two subunits: HIF-1α and HIF-1β (aryl hydrocarbon receptor nuclear translocator), whose pathway is targeted to control angiogenesis via the silencing of prolyl hydroxylase domain-containing protein 2 (PHD2) (Fig. 1B) [1, 18]. HIF-1α expression is tightly regulated by oxygen tension. PHD2 hydroxylates the key proline residues of HIF-1α, which targets HIF-1α for destruction via the ubiquitin-proteasome pathway, in normoxia. Under hypoxic conditions, HIF-1α accumulates leading to its stabilization and it partners with HIF-1β to bind to the HIF response elements on its target genes to turn on transcription of genes important to vasculogenesis, such as vascular endothelial growth factor (VEGF), erythropoietin (EPO), stromal-derived factor 1 (SDF1), leptin, and inducible nitric oxide synthase (iNOS) [11, 18]. The siRNA against PHD2 that blocks the binding of PHD2 with HIF-1α promotes the expression of angiogenic proteins via the hypoxia pathway. We hypothesized blocking of the bone differentiation pathway via siRNA against GNAS1 (siGNAS1) and the hypoxia pathway via siRNA against PHD2 (siPHD2) would affect MSC proliferation and expression of collagen, osteopontin, and alkaline phosphatase (ALP).

We therefore determined (1) whether the administration of siGNAS1 and siPHD2 in MSCs would promote osteogenic phenotype (cell proliferation and collagen, osteopontin, and ALP expression); (2) the dose-dependent effects of siGNAS1 on MSC differentiation to osteogenic phenotype (ALP assay) and the concentration of siGNAS1 needed for the in vivo bone formation study; and (3) whether the two siRNAs promote the formation of bone in vivo (bone volume and density with time).

## Materials and Methods

**Fig. 2A–B:**
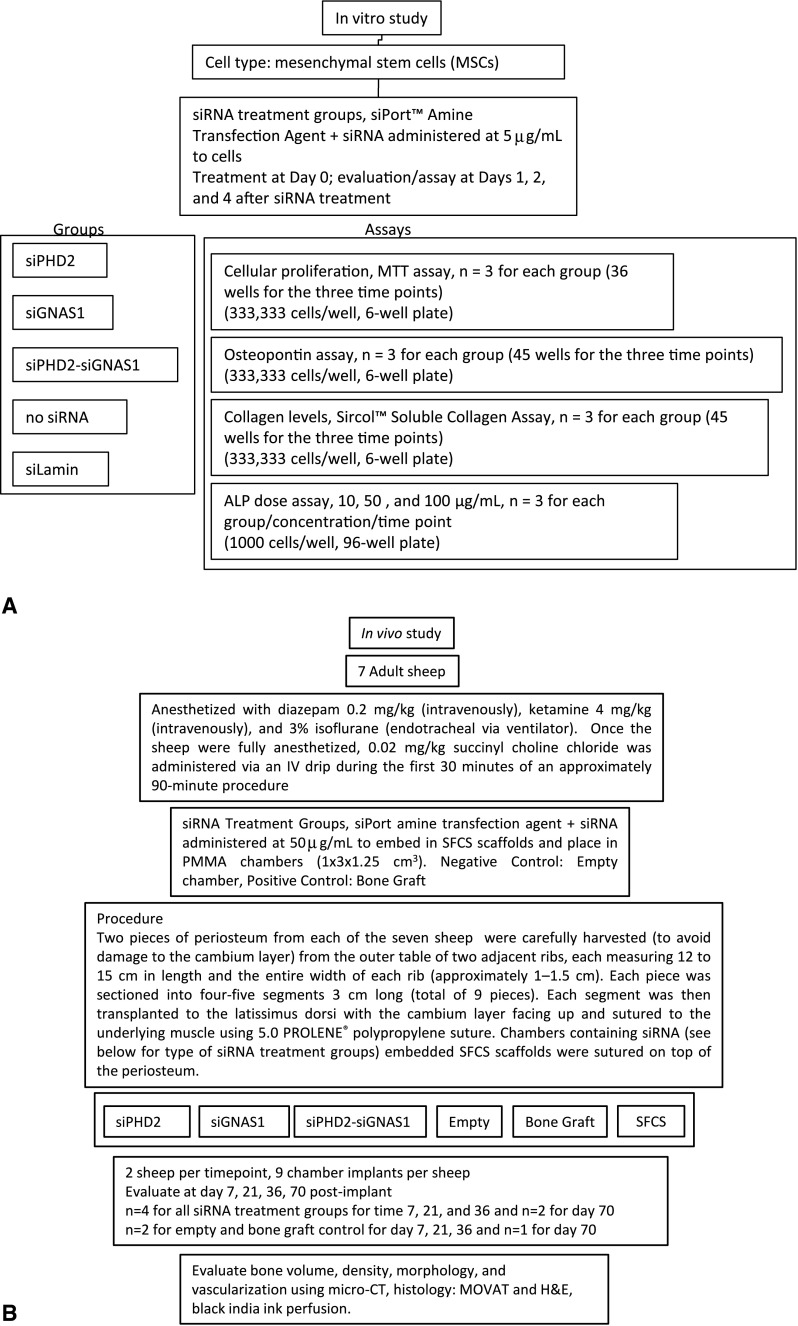
Flow diagrams show the setup of the (**A**) in vitro and (**B**) in vivo studies. The in vitro study involved the transfection of MSCs with siGNAS1 and siPHD2 and evaluation of cell proliferation, collagen expression, osteopontin expression, and dose-dependent ALP expression. The in vivo study involved embedding either siGNAS1 or siPHD2 or both in SFCS scaffolds and implanting them over periosteum engrafted on the latissimus dorsi muscle to fabricate ectopic bone.

This study was designed to investigate bone differentiation in SFCS scaffolds via the administration of siRNA in in vitro and in vivo assays (Fig. 2). The effect of siGNAS1 dosage on MSC differentiation to bone phenotype was determined using ALP assay (n = 3/treatment group/concentration/time point). The SFCS scaffolds were embedded with either a single siRNA or both together and bone volume and density with time were determined using micro-CT in an in vivo sheep model (n = 4/treatment group/time point, except at Day 70 [n = 2]).

For in vitro transfection with siRNA, human MSCs (ScienCell™ Research Laboratories, Carlsbad, CA, USA) were maintained in MSC medium, supplemented with 5% fetal bovine serum, 1% MSC growth supplement, and 1% penicillin/streptomycin solution. MSCs were incubated at 37° C in a humidified chamber containing 5% CO_2_. MSCs were seeded in six-well tissue culture plates at ~ 333,333 cells/well (n = 3 wells/treatment group). We had three time points with n = 3/group for cell proliferation, collagen expression, and osteopontin expression. Thus, there were 12 wells/time point for cell proliferation and 15 wells/time point for collagen and osteopontin expression. Overall, we had 36 wells for cell proliferation and 45 wells for collagen and osteopontin expression. Cells were allowed to adhere overnight. To transfect the cells with the siRNA, it was mixed with 5 μL Ambion siPort™ Amine Transfection Agent (Life Technologies, Grand Island, NY, USA) and incubated at room temperature for 10 minutes. Cells seeded in the wells were transfected with 5 μg/mL siGNAS1 (5′-GGCAACCAAAGUGCAGGACtt-3′; Applied Biosystems/Ambion Inc, Austin, TX, USA), siPHD2 (5′-AACGGGTTATGTACGTCATGT-3′), siGNAS1-siPHD2 (Qiagen Inc, Valencia, CA, USA), siLamin (5′-CUGGACUUCCAGAAGAACAtt-3′; Applied Biosystems/Ambion Inc), or nothing (control) at Time 0. After incubation for 1, 2, or 4 days, the cells were ready for 3-(4,5-dimethylthiazol-2-yl)-2,5-diphenyltetrazolium bromide (MTT) assay (cell proliferation), Western blot analysis (osteopontin), and collagen assay. To count the number of cells, we used an MTT assay from BioAssay Systems (Hayward, CA, USA). The reagents were incubated for 5 hours and measured by spectrophotometer.

For the osteopontin assay, the cells grown in the six-well tissue culture plates were lysed at the previously mentioned time points in 100 μL lysis buffer containing 2.5 μL phenylmethylsulfonyl fluoride, 2.5 μL sodium orthovandate, 2.5 μL protease inhibitor cocktail, and 1455 μL radioimmunoprecipitation assay buffer (all from Santa Cruz Biotechnology, Santa Cruz, CA, USA). Briefly, cell pellets were suspended in lysis buffer and kept on ice for 10 minutes. The suspensions were centrifuged at 4500 rpm for 10 minutes and supernatants were collected for analysis. Cell lysis aliquots equivalent to 50,000 cells were taken from each sample, mixed with similar volume of sodium dodecyl sulfate (SDS) buffer (BioRad, Hercules, CA, USA) containing 5% β-mercaptoethanol (Sigma-Aldrich Corp, St Louis, MO, USA), and heated at 100° C for 5 minutes. Samples were separated on a 10% SDS-polyacrylamide gel and then blotted for Western analyses. The nitrocellulose membrane was stained with antibodies specific for osteopontin (Santa Cruz Biotechnology). Intensities of various bands on Western blot films were scanned and analyzed using Gel-Pro Analyzer™ software (Media Cybernetics, Silver Spring, MD, USA). We normalized the band intensities from all time points to Day 1 control.

We performed the Sircol™ Soluble Collagen Assay (Biocolor Ltd, Carrickfergus, UK) on the siRNA-transfected MSCs. Absorbance was read at 550 nm using a microwell plate reader. Measurements were compared to standards and statistical analyses were performed appropriately.

For the ALP assay, we cultured MSCs in 96-well plates at 1000 cells/well overnight. siGNAS1 or siLamin at 10, 50, and 100 μg/mL (n = 3 wells/concentration) was added to 6-mm 75:25 SFCS scaffolds and placed over the MSCs in the wells. Scaffold only and no scaffold were used as control wells. We used the TRACP & ALP Assay Kit (Takara Bio, Inc, Otsu, Japan) to assess ALP in the siRNA-transfected MSCs. Absorbance was measured at 405 nm using a microwell plate reader.

**Fig. 3A–E:**
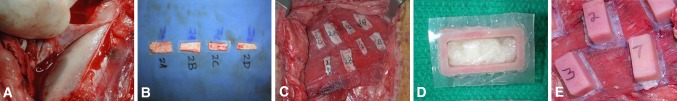
Photographs illustrate the surgical procedure for the in vivo sheep surgery. (**A**) Two pieces of periosteum (12–15 cm; width approximately 1–1.5 cm) were harvested from sheep ribs using a scalpel and periosteal elevator dissection, (**B**) cut into eight to nine 3-cm pieces, and (**C**) autografted over the latissimus dorsi muscle of the same sheep at 3 cm apart. (**D**) PMMA chambers, one chamber each for pure SFCS scaffold, bone graft, and empty as controls and two chambers each for siGNAS1-SFCS, siPHD2-SFCS, and siGNAS1-siPHD2-SFCS, were created and (**E**) implanted on top of the grafted periosteum on the latissimus dorsi muscle using PDMS to suture and 5.0 PROLENE^®^ suture.

Seven adult sheep raised and housed at The University of Texas M. D. Anderson Cancer Center’s Michael E. Keeling Center for Comparative Medicine and Research at Bastrop, TX, USA, were used for the in vivo study. Laws on animal experimentation were strictly followed and all protocols involving the animals were preapproved by the institutional animal care and use committee. Sheep were anesthetized with diazepam 0.2 mg/kg (intravenously), ketamine 4 mg/kg (intravenously), and 3% isoflurane (endotracheal via ventilator). Once the sheep were fully anesthetized, 0.02 mg/kg succinyl choline chloride was administered via an intravenous drip during the first 30 minutes of an approximately 90-minute procedure to reduce the occurrence and intensity of muscular spasms during surgery. Before surgery, the skin above the animal’s lateral chest wall was sheared, sterilized using iodine, and draped. The sheep was given an intramuscular injection of 1.0 g cefazolin and an incision was made overlying and parallel to the seventh or eighth rib to expose the two adjacent ribs (Fig. 3). Two pieces of periosteum from each of the seven sheep were carefully harvested (to avoid damage to the cambium layer) from the outer table of two adjacent ribs, each measuring 12 to 15 cm in length and the entire width of each rib (approximately 1–1.5 cm). The periosteum was lifted from the rib with a subperiosteal dissection. Each piece was sectioned into either four or five segments that were 3 cm long to make a total of nine pieces. Each segment was then transplanted to the latissimus dorsi with the cambium layer facing up and sutured to the underlying muscle using 5.0 PROLENE^®^ polypropylene suture (Ethicon, Inc, Somerville, NJ, USA).

A total of two sheep in each time point gave the study 85% power to detect an increase in maximal bone volume for siGNAS1-SFCS from Day 7 to Day 36 and 99% power from Day 7 to Day 70. It gave 99% power to detect an increase in maximal bone volume for siGNAS1-SFCS from Day 7 to Day 70 and Day 21 to Day 70. The study had 97% and 84% power to detect a siGNAS1-siPHD2-SFCS increase from Day 7 to Day 70 and Day 36 to Day 70, respectively.

We fabricated chambers (inner dimensions: 1 × 3 × 1.25 cm^3^) with polymethylmethacrylate (PMMA) and glued polydimethyl siloxane (PDMS) to the edges of the open end of the chamber. PDMS was then used to suture the chamber to the latissimus dorsi muscle. Chambers containing SFCS scaffolds with siGNAS1 (n = 4), siPHD2 [2] (n = 4), and siGNAS1-siPHD2 (n = 4) (75:25 blend [6] with 50 μg/mL of the siRNA) were implanted on top of the grafted periosteum over the latissimus dorsi muscle using the PDMS to suture the chamber to the muscle with 5.0 PROLENE^®^ suture. Each of the seven sheep had nine chamber implants (two for each of the four experimental groups and one for each control). Chambers were harvested after euthanasia (Beuthanasia 0.22 ml/kg intravenously) at Days 7, 21, and 36 with harvests from two sheep at each time point. Empty chambers (negative control, n = 2), chambers filled with bone graft (clinically relevant positive control, n = 2), and chambers filled with SFCS (n = 2) were controls for the time points above. Bone chips were used as fresh bone graft by dissecting bone from the ribs and crushing in a bone mill (Medtronic, Inc, Fort Worth, TX, USA). A similar procedure was used for implantation and harvest at Day 70, although the sample sizes for controls and experimental conditions were n = 1 and n = 2, respectively, since there was only one sheep harvested. To assess the extent of vascularization in SFCS scaffold after bone formation at the time of harvest, the lateral thoracic artery was perfused with india ink using an intravenous injection.

At the time of harvest, samples were preserved in 10% formalin and sent for micro-CT scans and then histologic staining. We performed micro-CT scans to assess bone formation in the chambers at the harvest time points at 92-μm sections/slices at the M. D. Anderson Small Animal Imaging Core facility using a Model RS-9 tabletop CT scanner (General Electric Medical Systems, London, ON, Canada). This scanner has a gantry that is rotated by a servo mechanism around the object bed, imaging the entire object in one pass. The gantry features a tungsten anode x-ray source, operated at 80 κVp and 450 iA, which is fixed opposite a detector composed of a cesium iodide scintillator and a charge-coupled camera. We used GE Healthcare’s MicroView software (Version 2.2) to analyze the regenerated bone. Regions of interest were outlined, three-dimensional isosurface images were generated, and bone volume and bone mineral densities were calculated [11]. The threshold was set to 100 for these measurements. Of the total four samples for the experimental groups and two samples for the control groups, we selected one image we believed representative (see below).

Subsequent to micro-CT imaging, samples were demineralized and dehydrated using a series of ethanol solutions and embedded in paraffin. Serial sections (4 μm) were cut, set on glass slides, and baked at 56°C overnight. Different sections from each sample were then deparaffinized, rehydrated, and stained with hematoxylin and eosin (H&E) or Movat’s pentachrome and imaged with bright-field light microscopy (Olympus IX50; Olympus America Inc, Center Valley, PA, USA).

Two-way ANOVA with Bonferroni post hoc test was conducted to determine the effect of the two siRNAs (independent variables) by assessing differences in dependent variables with time (independent variable): cell number, collagen expression, and osteopontin expression. Dose (independent variable)-dependent effects on ALP expression (dependent variable) with time (independent variable) were assessed using two-way ANOVA with Bonferroni post hoc test. One-way ANOVA with Tukey post hoc test was used to assess differences in bone volume (dependent variable) and bone density (dependent variable) with time (independent variable) among the siRNA treatment groups (independent variables). Our primary outcome was the bone volume measured by micro-CT scan. All data are reported as mean ± standard error of the mean (SEM) with the number of samples averaged per group (n) given in parentheses for each condition. We used GraphPad Prism^®^ 5 (GraphPad Software, La Jolla, CA, USA) to determine differences in cell proliferation and protein expression in vitro and bone volume and bone density in vivo between different groups (type of siRNA) with time. The sample size for bone volume and bone mass density measurements at Day 70 for SFCS controls was n = 1 and was not used for comparisons. We performed post hoc power analysis to determine the power in the study using nQuery Advisor^®^ 7.0 (Statistical Solutions, Saugus, MA, USA).

## Results

**Fig. 4:**
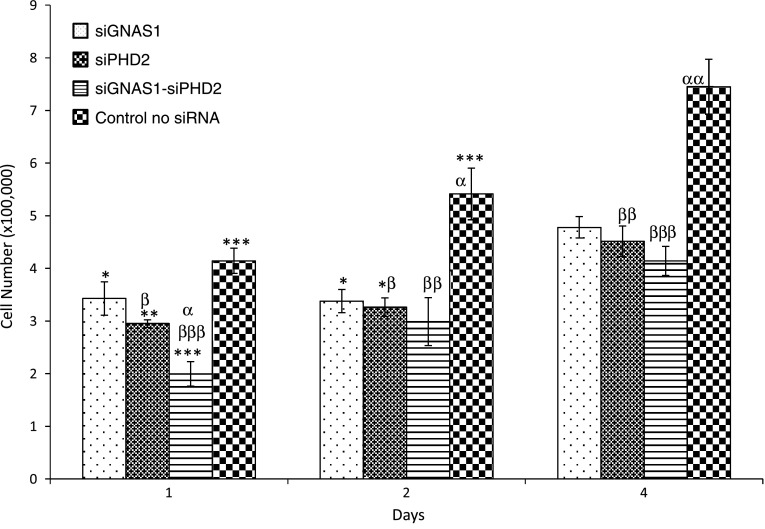
Cellular proliferation was measured using MTT assay for MSCs transfected with siGNAS1, siPHD2, siGNAS1-siPHD2, and no siRNA. MSC proliferation decreased with the administration of siRNA within 24 hours whereas there was an increase in cell number without any siRNA administration. Comparison among groups with time shows differences: * = p < 0.05, ** = p < 0.01, and *** = p < 0.001 compared to Day 4 in the same group; α = p < 0.05 and αα = p < 0.01 compared to GNAS1; β = p < 0.05, ββ = p < 0.01, and βββ = p < 0.001 compared to no siRNA within the respective time point. Values are expressed as mean ± SEM.

**Fig. 5:**
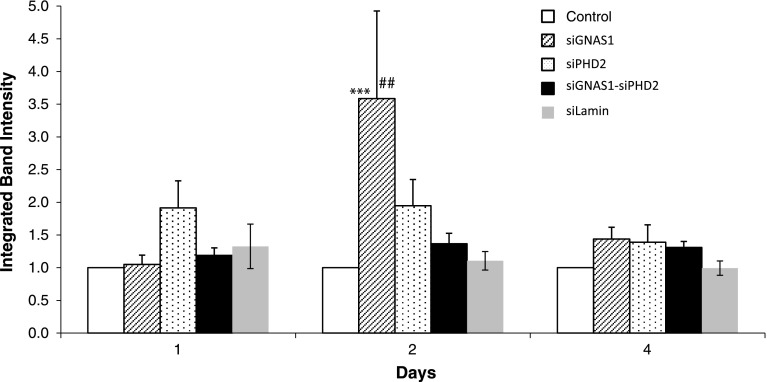
Collagen I expression was determined for MSCs transfected with siGNAS1, siPHD2, siGNAS1-siPHD2, siLamin, and no siRNA using Western blot. Maximal collagen I expression was observed at Day 2 in siGNAS1-treated cells. Comparison among groups with time shows differences: *** = p < 0.001 compared to Day 1; ## = p < 0.01 compared to Day 4. Values are expressed as mean ± SEM.

**Fig. 6:**
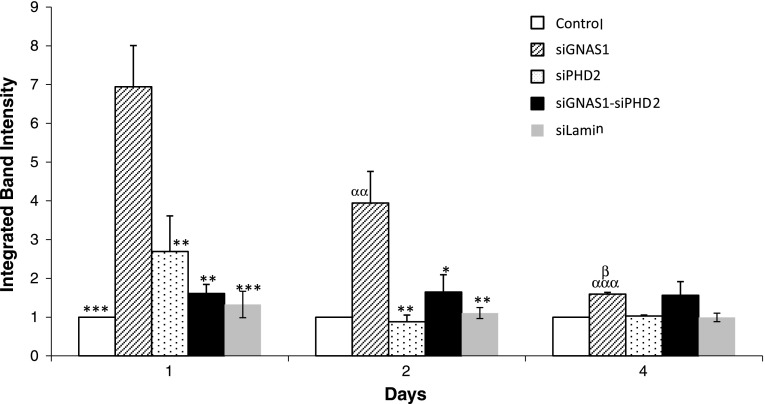
Osteopontin expression was determined for MSCs transfected with siGNAS1, siPHD2, siGNAS1 and siPHD2, siLamin, and no siRNA using Western blot. Osteopontin expression was maximal within 24 hours of siGNAS1 administration and decreased with time by Day 4, reaching the same expression level as siPHD2. The expression of osteopontin remained low for siPHD2-administered cells over the 4-day measurements. Comparison among groups with time shows differences: * = p < 0.05, ** = p < 0.01, and *** = p < 0.001 compared to siGNAS1 within the respective time point; αα = p < 0.01 and ααα = p < 0.001 compared to Day 1 siGNAS1; β = p < 0.05 compared to Day 2 siGNAS1. Values are expressed as mean ± SEM.

Cells proliferated from Day 1 to Day 4 with and without administration of siRNA, although the relative increase in cell number by Day 4 for cells with no siRNA was higher than for cells with siGNAS1 or siPHD2 or siGNAS1-siPHD2 (Fig. 4). For siGNAS1-administered MSCs, collagen I synthesis increased by Day 2 and then decreased by Day 4 (Fig. 5) as the siRNA was depleted. Osteopontin expression was maximal for siGNAS1-administered cells at Day 1 and was higher than that in cells administered siPHD2, siGNAS1-siPHD2, or siLamin (Fig. 6).

**Fig. 7:**
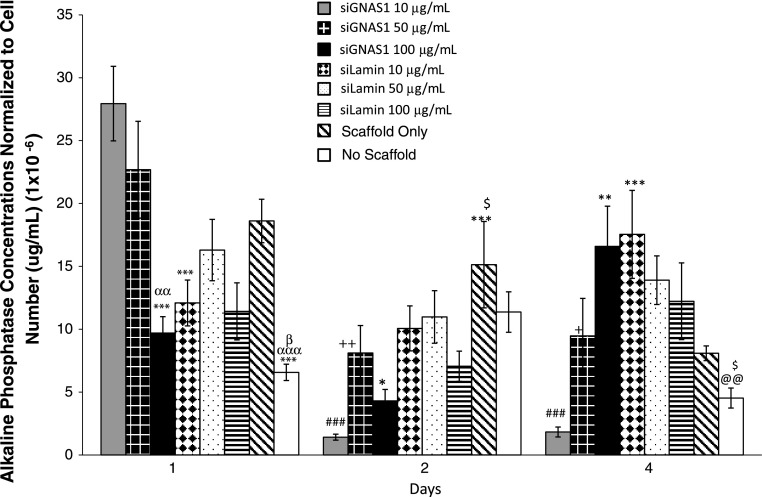
ALP assay was used to determine the effect of siGNAS1 concentrations at 10, 50, and 100 μg/mL and compared to siLamin (10, 50, and 100 μg/mL), scaffold only (no siRNA), and no scaffold (no siRNA) controls. The maximal ALP expression was observed for 10 and 50 μg/mL at Day 1, which decreased with time. The ALP expression for 100 μg/mL increased from Day 1 to Day 4. Comparison among groups shows differences: * = p < 0.05, ** = p < 0.01, *** = p < 0.001 compared to 10 μg/mL siGNAS1; ααα = p < 0.001 and αα = p < 0.01 compared to 50 μg/mL GNAS1; β = p < 0.05 compared to scaffold only; $$ = p < 0.01 and $ = p < 0.05 compared to 100 μg/mL siGNAS1; @@ = p < 0.01 compared to 10 μg/mL siLamin; ### = p < 0.01 compared to Day 1 for 10 μg/mL siGNAS1; ++ = p < 0.01 and + = p < 0.05 compared to Day 1 for 50 μg/mL siGNAS1. Values are expressed as mean ± SEM.

At Day 1, cells administered with 100 μg/mL siGNAS1 had lower ALP expression than the cells administered 10 and 50 μg/mL siGNAS1 (Fig. 7). ALP expression was higher for cells treated with siGNAS1 at concentrations of 10 and 50 μg/mL siGNAS1 on Day 1 as compared to untreated cells cultured on petri dishes with no scaffold and no siGNAS1. The presence of scaffold increased the ALP expression compared to no-scaffold controls, with no siGNAS1 administration in both cases. By Day 2, ALP expression decreased as compared to Day 1 for 10 and 50 μg/mL. There was no change in ALP expression from Day 2 to Day 4 for 10 and 50 μg/mL although it remained lower than Day 1 for both concentrations. At Day 4, administration of siGNAS1 at 100 μg/mL and siLamin at 10 μg/mL had higher ALP expression than administration of siGNAS1 at 10 μg/mL. Cells administered siLamin at 10 μg/mL and siGNAS1 at 100 μg/mL had higher ALP expression than no-scaffold/no-siGNAS1 controls.

**Fig. 8:**
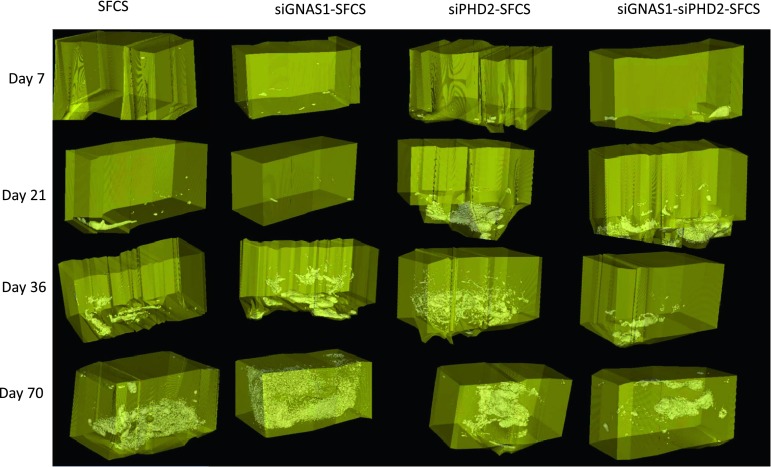
Micro-CT imaging of bone regeneration in pure SFCS scaffolds, siGNAS1-SFCS, siPHD2-SFCS, and siGNAS1-siPHD2-SFCS scaffolds at Days 7, 21, 36, and 70 after implantation shows bone formation in white. Bone formation is observed at the periosteum-chamber interface as early as Day 21 in SFCS, siPHD2-SFCS, and siGNAS1-siPHD2-SFCS. Bone filled into the chamber from Day 36 to Day 70 for siGNAS1-SFCS, siPHD2-SFCS, and siGNAS1-siPHD2-SFCS.

Representative micro-CT imaging of bone in the chambers showed differences in bone formation for the three siRNA groups (siGNAS1, siPHD2, and siGNAS1-siPHD2) from Day 7 to Day 70 (Fig. 8). Bone formation was observed at the periosteum interface (bottom of the chamber) as early as Day 7. By Day 21, there appeared to be an increase in bone formation at the interface and into the chamber in siPHD2-SFCS and siGNAS1-siPHD2-SFCS. There was further filling of the chamber with bone at Days 36 and 70 for all three conditions. Quantification of the bone volume indicated no difference in the empty chamber negative control with time and a decrease in bone volume of bone graft-positive controls with time (Table 1). There was also a difference in the bone volume between bone graft and empty chambers at Days 7 and 21. There was no change in bone mass density with time for bone graft and empty chambers (Table 1).

**Table 1:** Bone volume and bone mass density of bone graft and empty chambers

Variable	Day 7	Day 21	Day 36	Day 70
Bone volume (mm^3^)				
Bone graft	1436.0 ± 185.3	1036.8 ± 246.0^†^	531.7 ± 77.9*,^‡^	50.8*,^‡^,^‖^
Empty	135.0 ± 129.4*	195.2 ± 178.0^‡^	2.9 ± 2.8^§^	1.6
Bone density (mg/cm^3^)				
Bone graft	628.0 ± 6.1	646.2 ± 17.1	667.7 ± 42.3	708.1
Empty	165.3 ± 65.4	212.7 ± 6.5	116.4 ± 45.0	177.2

Values are expressed as mean ± standard error of the mean; * p < 0.0001, ^†^p < 0.01, as compared to bone graft at Day 7; ^‡^p < 0.001, as compared to bone graft at Day 21; ^§^p < 0.0001, ‖p < 0.01, as compared to bone graft at Day 36.

**Fig. 9:**
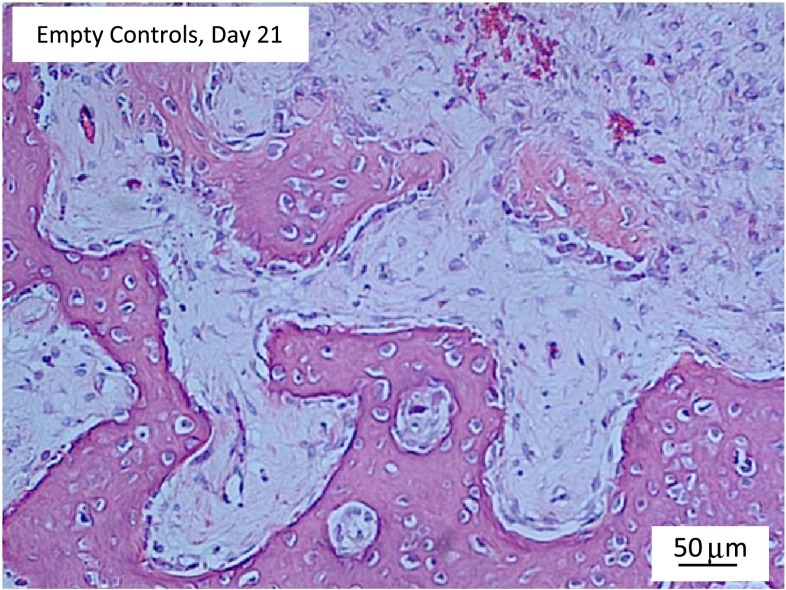
H&E staining of a 4-μm section of tissue harvested at Day 21 shows heterotopic bone formation at the muscle-periosteum interface with mineralizing bone in reddish-pink surrounding the newly forming marrow spaces in the middle.

**Fig. 10:**
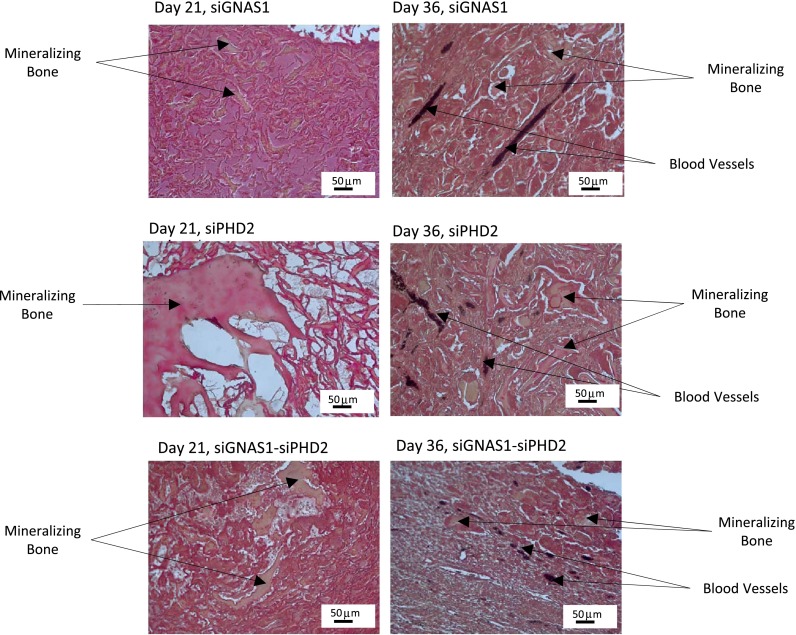
Movat’s pentachrome staining of 4-μm sections of tissue harvested at Days 21 and 36 shows mineralizing bone in deep yellowish-brown and mineralizing silk fibroin fibrils in pinkish-red. At Day 36, black india ink perfusion shows the vasculature in black for siGNAS1, siPHD2, and siGNAS1-siPHD2.

H&E staining of empty chamber microsections showed heterotopic bone formation within periosteum at the muscle-periosteum interface by Day 21 (Fig. 9). Histologic Movat’s pentachrome-stained microsections showed bone formation in SFCS scaffolds with siGNAS1, siPHD2, and siPHD2-siGNAS1 at Days 21 and 36 (Fig. 10).

**Fig. 11:**
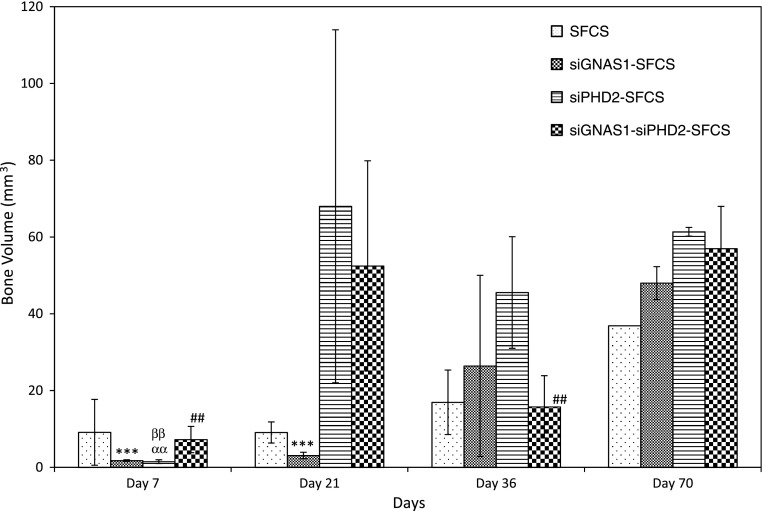
Bone volume was plotted against time to assess bone formation for SFCS scaffolds modified with siGNAS1, siPHD2, and siGNAS1-siPHD2. There was an overall increase in bone volume from Day 7 to Day 70 for siRNA-embedded scaffolds. For the siGNAS1 group, *** = p < 0.0001 compared to Day 70; for the siPHD2 group, αα = p < 0.01 compared to Day 36 and ββ = p < 0.01 compared to Day 70; for the siGNAS1-siPHD2 group, ## = p < 0.01 compared to Day 70. Values are expressed as mean ± SEM.

**Fig. 12:**
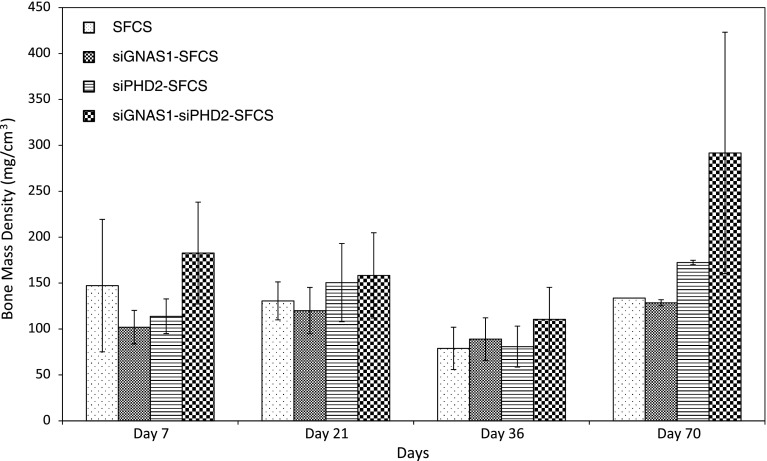
Bone mass density was plotted against time for SFCS scaffolds modified with siGNAS1, siPHD2, and siGNAS1-siPHD2. There was no difference in bone mass density among the groups. Values are expressed as mean ± SEM.

At Day 7, there was no difference in bone volume among the siRNA-embedded SFCS scaffolds (Fig. 11). At Day 21, siPHD2-SFCS and siGNAS1-siPHD2-SFCS tended to have higher bone volumes than SFCS and siGNAS1-SFCS. At Day 36, the maximal bone volume was observed for siPHD2-SFCS and was higher than Day 7. By Day 70, bone volume for siGNAS1-SFCS increased compared to Days 7 and 21, that for siPHD2-SFCS increased compared to Day 7, and that for siGNAS1-siPHD2-SFCS increased compared to Days 7 and 36. There was no difference in bone mass density among the siRNA-SFCS groups (Fig. 12).

## Discussion

siRNA allows the regulation of multiple cellular pathways. The Cbfa1 and HIF-1 pathways can be modulated to direct bone formation via siGNAS1 and siPHD2, respectively. We assessed the use of these siRNAs for modulating the bone formation pathway to provide a tool for promoting and directing the growth of functional bone for repair and reconstructive surgery applications. We determined (1) how the administration of siGNAS1 and siPHD2 in MSCs affects cell proliferation and collagen, osteopontin, and ALP expression; (2) how the dose of siGNAS1 affects ALP expression in MSCs as a bone differentiation marker and what concentration of siGNAS1 was needed for the in vivo bone formation study; and (3) whether the two siRNAs affect in vivo bone formation by measuring bone volume and density with time.

There were several limitations to this study. First, while we observed bone formation, the model provided no mechanical loading apart from the mechanical simulation from movement of the latissimus dorsi muscle. However, mechanical loading affects the bone formation with growth factor signaling in a clinical setting [9]. This type of prefabricated bone with no mechanical loading could have clinical applications. Such constructs could be moved as a bone tissue flap with the vascular supply from latissimus dorsi to a mechanically loaded defect site where the vascular supply could be connected but would have to be supported with metallic plates and screws initially until this prefabricated bone remodeled to specifications of the mechanical loading at the defect repair site. Second, we had only two experimental samples at Day 70 although we noted an increase in bone volume at Day 70 compared to Day 7. Third, siPHD2 and siGNAS1 both guide bone formation via complex and different intercellular mechanisms that were not explored in this study. Fourth, the overall concentration of the loaded siRNA in the SFCS scaffold and the rate of release of siRNA relative to the cells migrating into the scaffold, degradation of scaffold, differentiation of cells into osteogenic phenotype, and remodeling of new tissue in the sheep model need further investigation. Fifth, with the in vivo sheep model, no cells were seeded in the scaffold before implantation and the cell source for in vivo bone formation was limited to the periosteum. Since the vascular supply to the periosteum and the newly forming bone in the scaffold are from the latissimus dorsi muscle, cells would migrate in with the nutrient supply from the vasculature. Although it is well established that the cambium layer of the periosteum provides bone precursor cells, other cells that vascularize the scaffold may either migrate from the periosteum connective tissue or from the adjacent muscle.

We found osteogenic differentiation of MSCs can be mediated via siGNAS1 as indicated by increased expression of collagen I, osteopontin, and ALP. The administration of both siGNAS1 and siPHD2 slowed the proliferation of cells as compared to no-siRNA controls. Cellular proliferation was decreased by the presence of siRNA probably due to guidance toward the bone differentiation pathway. The cell cycle arrest in MSCs has been observed by others as the cells are directed toward a differentiation pathway [14]. In this study, collagen I expression was maximal at Day 2 after siGNAS1 administration in MSCs. Collagen I is the primary component of the bone extracellular matrix and its expression is regulated by the Cbfa1 transcriptional regulator [2, 5]. siGNAS1 promoted the expression of the proteins in the Cbfa1 pathway, while there was no effect of siPHD2 on the collagen I expression. Osteopontin, a phosphorylated sialoprotein, is found in the bone extracellular matrix [12]. Osteopontin expression would be expected to increase as a marker for the osteogenic phenotype as observed in this study within 1 day of exposure to siGNAS1 in MSCs. Osteogenic differentiation of MSCs marked by increase in Cbfa1 expression due to administration of antisense Gsα, which simulates inactivation of the GNAS1 gene similar to siRNA administration, has been studied [7]. Additionally, coadministration of siRNAs against GNAS1 and BMP2 reportedly accelerates the differentiation of human adipose tissue-derived stem cells toward osteogenic lineage [10].

siGNAS1 concentration affected ALP expression of MSCs. We performed the siGNAS1 dose assessment to determine the siGNAS1 concentration needed for the in vivo study. Lower concentrations of siGNAS1 exhibited higher ALP at Day 1, and as the siRNA depleted by Days 2 and 4, the ALP was reduced. While 10 and 50 μg/mL siGNAS1 resulted in maximal ALP expression after Day 1, the ALP expression decreased considerably for 10 μg/mL and intermediate effects were observed for 50 μg/mL as compared to 10 and 100 μg/mL. For the higher-concentration of siGNAS1, maximal ALP expression was not observed until Day 4, probably due to the effect of siRNA administration on cellular proliferation since the concentration values were normalized with overall cell number. The effect of siRNA in the cellular system on the cellular proliferation may have dominated the effect of siGNAS1 on the bone differentiation phenotype at short time points for 100 μg/mL. ALP detected at Day 1 was the lowest compared to 10 and 50 μg/mL, and as the cells proliferated by Day 4 and the siGNAS1 was utilized by the cells, the overall ALP expression increased for 100 μg/mL. Since the siGNAS1 targeted cellular proliferation and differentiation into an osteogenic phenotype in MSCs, the dose-dependent effects evident in this study were a function of the proliferation/differentiation balance that the cells try to achieve with siRNA administration to their system. Increased ALP activity, collagen I expression, and osteocalcin expression with increased in vivo bone formation have also been reported for Cbfa1 adenovirus-transfected MSCs [4].

In vivo data suggested an overall increase in bone volume from Day 7 to Day 70 in siGNAS1, siPHD2, and siGNAS1-siPHD2. At Day 21, siGNAS1-administered cells had a lower volume of bone, which increased by Day 70. An increase in bone volume was observed for siGNAS1, siPHD2, and siGNAS1-siPHD2 from Day 7 to Day 70. No change in bone density for any of the conditions indicated consistent bone density was maintained in the newly forming bone in the SFCS scaffolds with time up to 70 days.

The in vitro and in vivo effects of siPHD2 on bone formation were different. The sequence of siPHD2 used has been well tested in our laboratory to show it results in downregulation of PHD2 and upregulation of angiogenic factors such as iNOS [3]. It also promotes vacuole formation in endothelial cells at short time points (within 24 hours) and microvascular network formation by 96 hours. In this study, in vivo bone formation was aided by cells incoming from the periosteum and the adjacent latissimus dorsi muscle providing the vascular supply [16] to the periosteum. Thus, siPHD2 had the opportunity to act on other cells besides the bone precursors, although the siPHD2-driven vascular phenotype may support the regeneration of bone by supporting the nutrient supply to the growing bone.

The silencing RNA mechanism allowed us to control the fate of cell proliferation and differentiation by targeting the expression of multiple proteins via a single siRNA administration. Since siRNA affects the proliferative capacity of cells, for bone formation of critical sizes, it is important to consider the type of cells with respect to the type of siRNA, time of siRNA introduction, amount or concentration of siRNA with respect to the number of cells in the system, and sustained release of needed amount with time, so as to not to promote premature differentiation of cells without adequate proliferation to fill or repair a defect.
